# Enhancing Deep Edge Detection through Normalized Hadamard-Product Fusion

**DOI:** 10.3390/jimaging10030062

**Published:** 2024-02-29

**Authors:** Gang Hu, Conner Saeli

**Affiliations:** 1 Department of Computer Information Systems, SUNY Buffalo State University, Buffalo, NY 14222, USA; 2 Center for Computational Research, SUNY University at Buffalo, Buffalo, NY 14203, USA

**Keywords:** edge detection, Hadamard product, mutual agreement, salient, fusion, deep network

## Abstract

Deep edge detection is challenging, especially with the existing methods, like HED (holistic edge detection). These methods combine multiple feature side outputs (SOs) to create the final edge map, but they neglect diverse edge importance within one output. This creates a problem: to include desired edges, unwanted noise must also be accepted. As a result, the output often has increased noise or thick edges, ignoring important boundaries. To address this, we propose a new approach called the normalized Hadamard-product (NHP) operation-based deep network for edge detection. By multiplying the side outputs from the backbone network, the Hadamard-product operation encourages agreement among features across different scales while suppressing disagreed weak signals. This method produces additional Mutually Agreed Salient Edge (MASE) maps to enrich the hierarchical level of side outputs without adding complexity. Our experiments demonstrate that the NHP operation significantly improves performance, e.g., an ODS score reaching 0.818 on BSDS500, outperforming human performance (0.803), achieving state-of-the-art results in deep edge detection.

## 1. Introduction

In the realm of image processing, human visual perception heavily relies on shapes [1]. Contour-based shape features, which leverage object boundary information, offer a more intricate understanding of object shapes. Edge detection techniques play a pivotal role by extracting salient object boundaries, preserving the essence of an image, and filtering out unintended details. Perceptual edge features, classified as low-level features, play a crucial role in mid- and high-level visual analysis tasks, encompassing image segmentation, object detection, and recognition.

While traditional edge detection methods rely on low-level visual cues and handcrafted feature representations, recent research efforts have shifted toward deep learning models in the computer vision community. Convolutional Neural Network (CNN)-based approaches have risen to prominence, becoming the mainstream framework for image classification, object detection, semantic segmentation, and edge detection domains. Notable methods like DeepEdge [2], $N^{4}$-Fields [3], CSCNN [4], DeepContour [5], and HED [6] have significantly advanced edge detection performance. HED, a successful deep network framework, employs a holistically nested architecture, generating multiple intermediate edge side outputs (SOs) along the network pathway, which are then fused to produce the final edge result.

Despite the progress in deep edge detection approaches, a common drawback arises in the fusion step. This involves the challenge of balancing noisy edges and global contours within a single SO. The conventional fusion practice assigns optimal weights to an individual layer’s SO edge maps, treating all pixels from a single edge map equally. Therefore, this approach fails to distinguish between noise and fine data within a single SO, impacting the detection quality and accuracy. The network faces a dilemma in managing noisy edges while preserving global boundaries, making semantic edge detection a persistent challenge.

**Figure 1:**
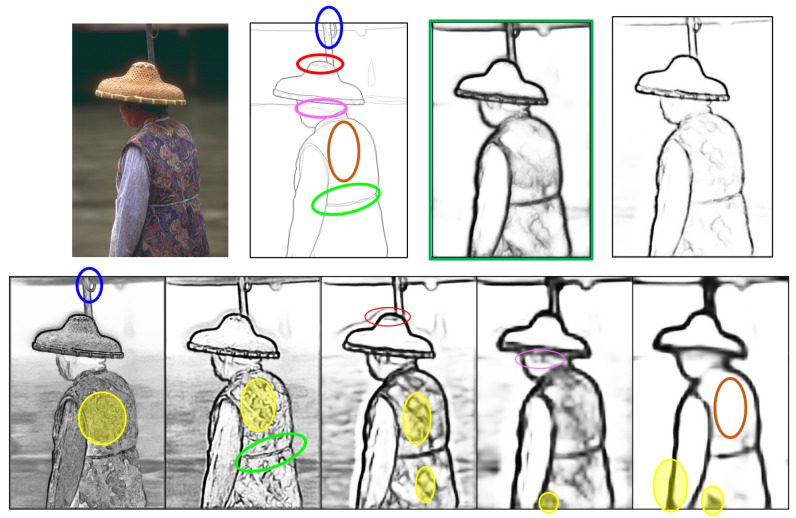
The issue of the existing approaches. The first row shows the original image, ground-truth edges, and the fused results from a typical existing approach and ours. The second row lists five side outputs from the left to right. The areas in blue, green, red, pink and brown circles on the ground truth match the corresponding areas in $SO_{1}∼SO_{5}$ respectively.

Figure 1 illustrates an input image along with its ground-truth edges, the results from an existing approach (in green box), and our results in the first row. The second row shows five SOs ($SO_{1}∼SO_{5}$ from left to right), each representing an edge map at a specific spatial scale. Finer-scale $SO_{1}$ and $SO_{2}$ exhibit thin contours and noise, while coarser $SO_{4}$ and $SO_{5}$ have fatty edges due to upsampling, lacking the necessary details. The ground truth is marked with circled areas indicating the best matches from different SOs. Here, the edges of the waistbelt from $SO_{1}$ (circled in green), the shape of the umbrella’s top spring areas from $SO_{2}$ (circled in blue), the texture patterns of the crown top of the conical hat from $SO_{3}$ (circled in red), and the hair boundary near the ear side from $SO_{4}$ (circled in pink) closely match the ground truth and should be fused into the optimal final result. However, the existing fusion process applies a single weight to each SO indiscriminately, without considering the edge importance. Consequently, when the network requires specific parts from a certain SO, all others from this SO are also included. In Figure 1, the portions shaded in yellow in $SO_{i}$ would appear in the final output if the corresponding circled parts are considered. As a result, the output often contains noise and thick edges while missing some key boundaries. To tackle this issue, a complicated deeper network is required to learn accurate pixel-wise importance. However, when neural networks deepen, gradient vanishing can occur during backpropagation, where gradients become extremely small and lose impact on updating earlier layers, resulting in slow or no learning. Although skip connections (e.g., in ResNet) help mitigate this issue, deeper networks may still face problems, like degradation, slow convergence, parameter optimization difficulties, and overfitting [7].

To address the aforementioned issue, the research question arises: instead of uniformly applying a single weight to the entire SO edge map, can we selectively choose important details from each edge map for fusion? Rather than opting for a deeper and more complex network structure with pixel-wise weights, which poses training challenges, we propose the normalized Hadamard-product (NHP) operation. This operation enhances the fusion process by incorporating more edge maps without increasing the network complexity. These additional edge maps are subsets of existing SOs, extracted through the NHP operation. The NHP operation, a multiplicative process, facilitates the promotion of agreed-upon features while suppressing disagreements. By applying the NHP operation on multiple SOs, agreed edge feature maps are generated, named as Mutually Agreed Salient Edge (MASE) maps with varying levels of importance. Besides the existing multi-scale SO feature maps, MASE maps provide additional enriched hierarchical structure that distinguishes between local and global edges. During the fusion stage, this increased granularity of edges offers more options for the network to produce better results. For example, in Figure 1, by applying NHP operations, the circled areas would be extracted from different SO edge maps and subsequently fused into the final result without incorporating many noisy edges (shaded in yellow). The main contributions of the proposed enhancement approach include the following:

- The generation of the NHP resulting in additional MASE maps containing key scale-invariant features that reflect true object boundaries;
- Combining MASE maps produced by the NHP with existing SOs provides more hierarchical structure for fusion operation;
- The network is guided to learn semantic edge boundaries by deep supervision on MASE maps and the original SOs;
- Using the NHP operation for strong edge selection does not increase the network complexity;
- Experiments conducted on the widely used BSDS500 [8] and NYUD [9] datasets demonstrate that the proposed framework outperforms other methods.

The rest of this paper is organized as follows. Section 2 introduces the related work. Section 3 presents the proposed NHP method and MASE maps for edge detection in detail. The experimental results and comparisons are presented in Section 4. Finally, the discussion and conclusions are given in Section 5 and Section 6, respectively.

## 2. Related Work

Edge detection is a fundamental task in image processing, playing a vital role in high-level image understanding and computer vision. There have been numerous edge detection approaches in the literature. In the early days, intensity gradient-based techniques were prevalent. Those algorithms often involved convolving images with a Gaussian filter for smoothing, followed by the application of handcrafted kernels to identify pixel intensity gaps representing edges. Classic methods such as the Canny detector [10], Sobel detector [11], and zero-crossing [12] fall within this category. However, these pioneering methods heavily relied on manually designed low-level features. As we know, low-level features are often sensitive to noise, illumination changes, and other variations. Often, an edge detector may produce false positives or miss important edges in noisy images or under varying lighting conditions. For instance, edge detectors may produce different results for the same object at different scales or orientations. Low-level features lack the ability to capture semantic information or object-level understanding. It may detect object edges, but it might not understand what those objects represent.

In response to the limitations posed by low-level features, prior to the advent of deep learning, researchers leveraged machine learning algorithms and probabilistic theories to devise various handcrafted mid–high-level features for visual analysis tasks. These manually crafted features incorporated both top–down prior knowledge and bottom–up visual clues, characterized by clear definitions and intuitive interpretations. To increase the robustness of edge features, in the Statistical Edges approach [13], edges were obtained from the learned probability distributions of edge-filtering responses. To reduce the data noise, in [14], a fuzzy rules-based filtering system was proposed to perform edge detection with reliable performance. Martin et al. [15] crafted the posterior probability (Pb) of boundary features, derived from changes in local visual cues (brightness, color, and texture), which were then input into a classifier for edge detection. To make Pb features equipped with global semantics, a globalized gPb [8] was introduced using standard Normalized Cuts [16]. Promoting object-level understanding is a key for better performance in visual tasks. Lim [17] proposed handcrafted Sketch tokens to represent mid-level information. In Ref. [18], instead of directly using low-level cues (color and gradients), a patch-based mid-level structural representation was summarized to detect high-quality edges with the help of random decision forests. The PCPG model [1] drew inspiration from perceptual origination, formulating gestalt laws to define and detect generic edge tokens (GETs). These handcrafted approaches with mid- to high-level semantics consistently outperformed pioneer methods. However, handcrafted features have several drawbacks. They are often designed based on smaller datasets or domain knowledge, which usually would lack robustness to handle data variations. The edge detection solutions based on the handcrafted features usually are designed for the specific tasks without the generality for a wide range of application domains.

The introduction of deep neural networks has transformed the landscape of edge detection. Convolutional Neural Networks (CNNs) emerged as powerful tools for automating feature extraction and learning intricate patterns directly from raw data. It redefined the standards for edge detection, surpassing the performance of handcrafted feature engineering. Because edges are derived from local neighboring pixels, $N^{4}$-Fields [3] combined CNNs with the nearest neighbor search for edge detection. To boost object-level understanding, deep contour [5] partitioned object contours into subclasses and fit each subclass based on a learned model. In another approach [4], DenseNet was employed in pixel-wise deep learning to extract feature vectors, using an SVM classifier to categorize each pixel into the edge or non-edge class. In this way, the edge detection was transformed into an object classification task. HED [6] utilized VGG16 as its backbone network for feature extraction, generating an edge map from each convolutional block to construct a multi-scale learning architecture. The multi-scale SO edge maps representing local-global views were fused as the final result. RCF [19] enriched each SO edge map with an extra convolutional layer, which improves HED’s performance. CED [20] added a backward-refining pathway to make the edge boundaries thinner by using a non-maximum suppression loss function. In the bidirectional cascade network (BDCN) [21], also designed on top of VGG for edge extraction, a scale enhancement module generated multi-scale features and detected edge contours at different scales using dilated convolution layers. BDCN employed a cascade structure composed of a forward stream and a backward stream. This structure allows for bidirectional information flow and enhances the network’s ability to capture edge information. However, a common drawback in these approaches is observed in the fusion step: edge maps from earlier layers contain more noise and lack global semantic information, with feature data in the same edge map sharing the same weight and having equal importance in fusion.

To address this fusion drawback, one must either improve the quality of the intermediate edge maps or design a better fusion block. An encoder–decoder network structure is feasible to improve the intermediate SOs. A decoder structure of U-Net [22] was used in [23] to incorporate global information into shallow features. However, a recent study [24] suggested that semantic information gradually decays as it is fused downward in U-Net structures. An edge detection approach [25] based on U-Net performed poorly on the BSDS500 dataset. To avoid important edge features vanishing along the deep convolutional operation, a network with two parallel skip connections was designed in [26]. The feature maps generated at each block were fed to a separate upsampling network to create intermediate SO edge maps. Elharrouss et al. [27] used refined batch normalization with learnable affine parameters to make the intermediate SOs less noisy around the edges. At the end of the network, these features were fused to generate a better edge map. CATS [28] attempted to improve the fusion issue with a context-aware fusion block (coFusion) plugged into an existing edge detection network. This fusion block aggregates the complementary merits of all edge maps, suppressing the nearest neighbor around the edges to obtain crisp edge boundaries. However, it is an add-on extension to existing frameworks rather than a complete end-to-end solution to the edge detection task.

Transformer-based frameworks [29,30,31], successful in the NLP domain, inspired the use of vision transformers like ViT [32] for various visual tasks. In [33], a vision-transformer-based model for edge detection, EDTER, was proposed. It consists of two-stage encoders (global and local) followed by a Bidirectional Multi-Level Aggregation decoder to achieve high-resolution features. The global and local cues are combined by a feature fusion module and fed into a decision head for edge prediction. More recently, a diffusion probabilistic model (DPM)-based edge detection approach was proposed in [34], including an adaptive FFT-filter and uncertainty distillation strategy. DiffusionEdge is able to directly generate accurate and crisp edge maps without any post-processing. However, those new networks are computationally expensive in terms of the complexity and number of parameters. For example, EDTER requires 900+ GFLOPs (Giga FLoating-point Operations Per Second).

To reduce the network complexity, a Lightweight Dense Convolutional (LDC) [35] neural network was proposed for edge detection. LDC using only 674 k parameters reaches a similar performance when comparing with heavy architectures (models with about 35 million parameters). Based on LDC, the TEED (tiny and efficient edge detection) model [36] uses even fewer parameters (58 k). It makes the model easy to train and quickly converges within the first few epochs while producing crisp and high-quality edge maps.

In summary, modern edge detection methods rely on deep network-based approaches for their superior performance. However, they encounter a common issue: the use of a single fusion weight for the entire edge map, resulting in noise and thick edges in the final output when combining multi-scale edge maps (as illustrated in Figure 1). Despite the introduction of more effective backbone networks to enhance the quality of intermediate SO edge maps, these solutions often incur high computational costs due to their complexity and large parameter size. In this study, we propose an effective approach to enhance edge detection performance without increasing network complexity.

## 3. Our Approach

Our approach aims to address the global and local feature-balancing problem in the fusion step by employing an efficient Hadamard-product operation. Here, we first explore the Hadamard product and then explain its application within the Mutually Agreed Salient Edge (MASE) framework for edge map enhancement. The outputs generated by the MASE undergo gradual refinement through the loss functions in an end-to-end manner. Utilizing the normalized Hadamard product (NHP) following a backbone network structure, such as VGG-16, results in the outcomes of more accurate edge maps.

### 3.1. Hadamard Product

The original Hadamard product constitutes an element-wise multiplicative operation that takes two matrices of the same dimensions as operands, producing another matrix of identical dimensions (see Equation (1)).

(1) $$\begin{array}{c} A⊙B=C\\ \left[\begin{array}{cccc} a_{11} & a_{12} & \cdots & a_{1n}\\ a_{21} & a_{22} & \cdots & a_{2n}\\ \vdots & \vdots & \ddots & \vdots \\ a_{m1} & a_{m2} & \cdots & a_{mn} \end{array}\right]⊙\left[\begin{array}{cccc} b_{11} & b_{12} & \cdots & b_{1n}\\ b_{21} & b_{22} & \cdots & b_{2n}\\ \vdots & \vdots & \ddots & \vdots \\ b_{m1} & b_{m2} & \cdots & b_{mn} \end{array}\right]=\left[\begin{array}{cccc} c_{11} & c_{12} & \cdots & c_{1n}\\ c_{21} & c_{22} & \cdots & c_{2n}\\ \vdots & \vdots & \ddots & \vdots \\ c_{m1} & c_{m2} & \cdots & c_{mn} \end{array}\right],\\ c_{ij}=a_{ij}\cdot b_{ij},i\in \left[1,m\right],j\in \left[1,n\right]. \end{array}$$

**Figure 2:**
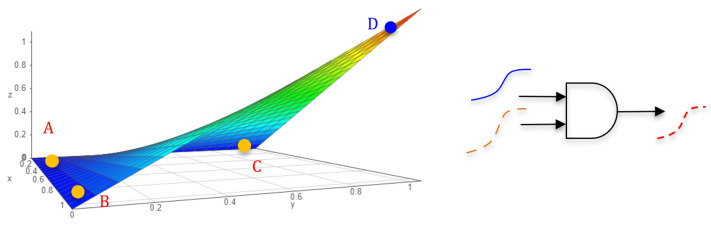
Normalized Hadamard-Product operation. The plot of element-wise multiplication z=x·y, where *x* and *y* are in [0, 1]. It acts essentially as an AND gate of 2 input signals.

When the values $a_{ij},b_{ij}$ of both input matrices (*A* and *B*) are either zeros or ones, the element-wise multiplication $c_{ij}=a_{ij}\cdot b_{ij}$ naturally yields a structure akin to an AND gating system, where a result value $c_{ij}$ from matrix *C* is one only when both input element values are ones. If the values of both input matrices are normalized within the range of [0, 1], the resulting matrix values also fall between 0 and 1. A small resulting value indicates that at least one of the inputs is very small, while a value close to 1 signifies that both input values are substantial. This reflects the semantic meaning of the degree of the element-wise agreement, indicating a significant agreement when both inputs are substantial. Figure 2 illustrates a 3D space (*x*, *y*, and *z*), where the plot represents a normalized Hadamard-product (NHP) function z=x·y because both *x* and *y* are normalized within the range of [0, 1]. The plotted color surface intuitively reveals the mutual agreement level between *x* and *y*.

For instance, point A’s *z* value is minimum because both A’s *x* and *y* are small. The *z* values of points B and C are also minimal because either *x* or *y* of both points is small. Only point D’s *z* value is maximized due to both its *x* and *y* being large. The NHP operation enhances the *z* value when mutually supported by the *x* and *y* inputs. This AND-like gating operation of the NHP acknowledges the salience when both inputs exhibit salience. In the deep neural network-based edge detection framework computation, considering the state-to-state computation A⊙B, A (representing the local SO feature map) and B (representing the global SO feature map) are cross-checked to determine the element-wise agreements on the edge boundaries. Generally, strong edges from different scales easily find agreement, while noisy edges from the local feature are minimized as they typically lack endorsement from the global view. In this work, this property of the NHP is harnessed in the deep neural network to extract better edge boundaries from both local and global feature maps.

It is noteworthy that the NHP operation is a differentiable function with a smooth surface (Figure 2). This characteristic is particularly desirable for gradient-based convex optimization within deep learning networks.

### 3.2. Mutually Agreed Salient Edge (MASE) Framework

**Figure 3:**
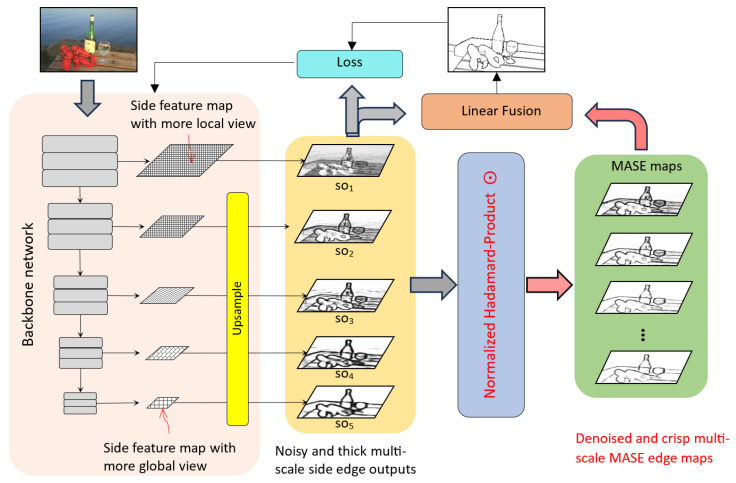
Overall framework. The Mutually Agreed Salient Edge (MASE) framework has two components: the backbone network with 5 intermediate side outputs and a fusion module that consolidates these side outputs and MASE maps into the ultimate edge result.

In the existing deep network-based edge detectors, the final edge results are fused by multiple SOs produced from the backbone network. The more and better selection options the fusion process has, the better the edge result anticipated is. However, without increasing the network complexity, the number of the SO is fixed. As explained in Section 3.1, the NHP operation is able to promote mutually agreed information while suppressing disagreements. In this work, we utilize NHP’s property to extract additional fusion candidates with enriched hierarchical edge structures. Figure 3 illustrates the overall architecture of the MASE network, consisting of two key components: the backbone network (VGG16) with 5 intermediate SOs and a fusion module that consolidates these SOs and MASE maps into the ultimate edge result. The network gradually reduces the scales of feature maps while increasing the data channels through the network pathway. To obtain a single channel SO, a 2D convolution layer is applied, where multiple channel data are merged into one channel. The higher-scale SOs from earlier convolution layers have more local edge features, including noisy signals and unnecessary small edges, typically representing texture details or noise, which should not be treated as object contours. Conversely, the lower-scale SOs often contain coarser and more global boundaries as they are upsampled from smaller feature maps. There are also some edges appearing across both high-scale and low-scale SOs. They represent object contours, spatial boundaries, or edge details with strong pixel gradients. These features, endorsed by multiple scale views, provide crucial information about the object shape and scene semantics. Our objective is to identify and extract the mutually agreed edge salience as the foundation for enhancing the final edge map.

To accomplish this, an additional network layer, the normalized Hadamard-product operation, is integrated into our framework. This operation involves normalization, Hadamard product, rescaling, and linear combination processes. The subsequent sections provide detailed explanations of each of these processes.

#### 3.2.1. Normalization

To ensure that the Hadamard operation functions as an AND-like gate, amplifying mutually agreed edge salience features while suppressing disagreed edges, data normalization becomes essential. The data from the side outputs have a broad range. For instance, the data from the first side output ($so_{1}$) fall ∈(−200,500). As explained in Section 3.1, the Hadamard operation may not perform as an AND gate when the values are outside of [0,1]. To address this, a sigmoid function is employed to normalize the data:

(2) $$f_{i}=\frac{1}{1+exp(-so_{i})}$$

where $so_{i}$ is a side output, i∈[1,5], and its values could be in (−∞, +∞); the data range of the normalized $f_{i}$ is ∈[0,1].

#### 3.2.2. Hadamard Product and Rescaling

**Figure 4:**
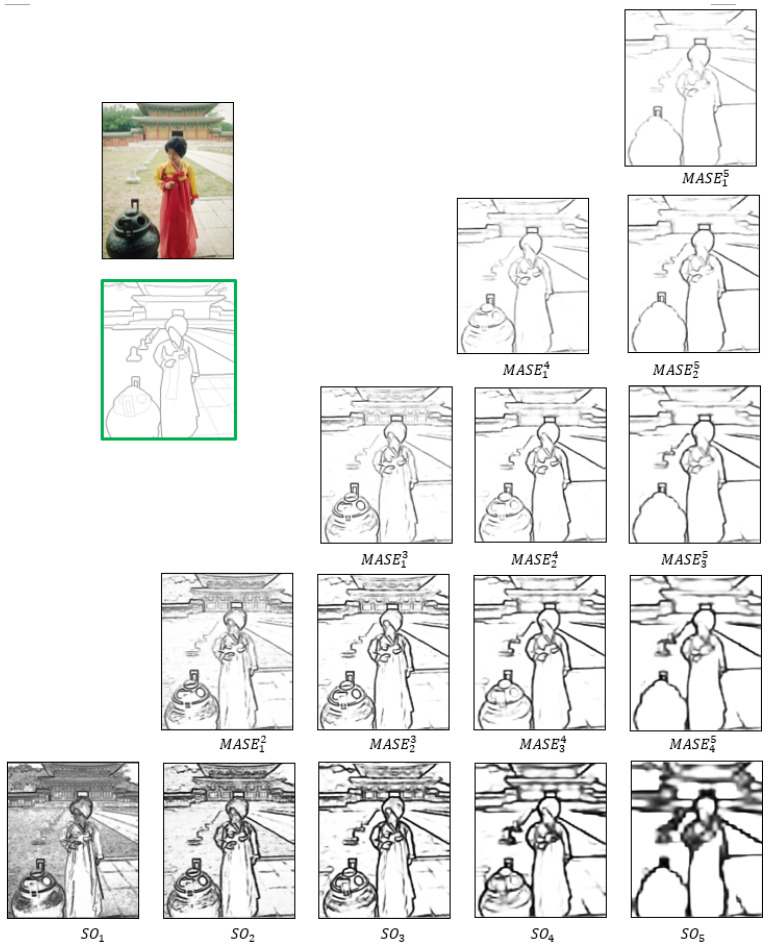
Hierarchical MASE maps for edge enhancement. The bottom row contains 5 side outputs ($so_{i=1∼5}$) while the $MASE_{j}^{k}$ maps (j=1∼4,k=2∼5,k>j) are above the bottom row.

The Hadamard (element-wise)-product operator is employed to generate MASE maps by operating on multiple normalized SOs. The computation of MASE maps begins with consecutive neighboring SOs as follows:

(3) $$MASE_{j}^{k}=Logit_{k}\left(\prod_{i=j}^{k}f_{i}\right),j\in \left[1,4\right],k\in \left[2,5\right],k>j$$

where ∏ represents the Hadamard product, $f_{i}$ is a normalized SO from Equation (2), and $Logit_{k}\left(\cdot \right)$ is the inverse function to the sigmoid in Equation (2). This inverse function rescales the values back to the original data range of $so_{k}$. Consequently, the resulting $MASE_{j}^{k}$ is the subset from $so_{j}∼so_{k}$. Figure 4 illustrates examples of the computed MASE maps, with the bottom row containing five SOs. Above them, all the $MASE_{j}^{k}$ are presented row by row, culminating in the top $MASE_{1}^{5}$, which includes all $so_{i}$. In comparison with $so_{i}$, the local edge details in corresponding MASE maps are significantly reduced. The reduction occurs because only mutually agreed salient values are preserved, diminishing disagreements between adjacent SO features. Meanwhile, only salient boundaries survived when the MASE map is computed from more low-scale SOs. For example, $MAES_{1}^{2}$ is the mutual agreement between $so_{1}$ and $so_{2}$ and preserves important edge boundaries with significantly reduced noise.

Among all the $MASE_{j}^{k}$, $MASE_{1}^{2}$ contains the most local edge details, while $MASE_{1}^{5}$ encapsulates the denoised and crisp global object boundaries, as only edges agreed upon by all SO feature maps are retained. Overall, MASE maps not only have better edge quality and less noise than SOs (see Figure 4) but also offer additional intermediate views with enriched hierarchies of local and global perspectives. It is important to note that this process does not necessitate additional network parameters, preventing an increase in complexity.

#### 3.2.3. Linear Combination

MASE maps effectively segregate local details and global boundaries into distinct hierarchies with finer granularity. The rescaled values of these maps align with the data range of the side outputs. Equation (4) outlines the aggregation of side outputs and MASE maps to enhance the final results:

(4) $$P_{fuse}=\sum_{e=1}^{E}P_{e}\cdot W_{e}$$

Here, *E* is a set of generated edge maps including side outputs and MASE maps $E=\{so_{1}∼so_{5},MASE_{j}^{k}\}$. Each $P_{e}$∈E is weighted by $W_{e}$. Given that *E* comprises finely separated local and global edges, the result edge prediction *P* selectively incorporates proper local and global edge feature values based on the assigned weights. Equation (4) is indeed the fusion process in our method, which is simple yet effective. Note, the weights $W_{e}$ are learned through the network training process under the supervision of the loss functions.

### 3.3. Network Training and Loss Functions

Here, we briefly explain the loss functions and training process for the proposed MASE framework. As we can see from Figure 3, each SO in this network and the produced MASE maps can be trained with layer-specific side supervision. The final fused result is supervised at the loss layer as well. The overall loss is formulated as:

(5) $$L=\sum_{e=1}^{E}L_{e}\left(P_{e},Y\right)+L_{fuse}\left(P_{fuse},Y\right)$$

where $E=\{so_{1}∼so_{5},MASE_{j}^{k}\}$; the $L_{e}$ and $L_{fuse}$ functions compute the difference between the edge prediction *P* and the edge label *Y*:

(6) $$L_{(}p_{i},y)=-\alpha \sum_{i\in Y_{-}}log\left(1-p_{i}\right)-\beta \sum_{i\in Y_{+}}log\left(p_{i}\right)$$

where $p_{i}$ is the edge prediction and $Y_{+}$ and $Y_{-}$ denote edge and non-edge pixels, respectively. Both $\alpha =\lambda \cdot |Y_{+}\left|/(\right|Y_{+}\left|+\right|Y_{-}\left|\right)$ and $\beta =|Y_{-}\left|/(\right|Y_{+}\left|+\right|Y_{-}\left|\right)$ are used to balance the training samples and are controlled by the hyper-parameter λ. It is also worth noting that all the steps in the fusion module (sigmoid, Hadamard product and rescaling, and linear combination) are differentiable at all points, which means that no additional adjustment is required for parameter learning during network backpropagation.

In summary, our approach utilizes the NHP operation to extract MASE edge maps without increasing the network complexity. These extra edge maps contain mutually agreed edge information over multiple existing SOs and are strong evidence of object contours. These MASE maps enrich the set of edge map candidates, which enhances the final edge quality in the fusion process.

## 4. Experiments

We conducted our experiments with ablation studies on various MASE maps. Subsequently, the fused results are evaluated on two widely used public benchmarks. To showcase the effectiveness of our proposed approach, we also conduct comparisons with other state-of-the-art methods. Finally, subjective evaluation results are presented. This framework is programmed in Python 3.10 using PyTorch. All the experiments were conducted on an Intel i7-8700 CPU running Ubuntu 18.04, equipped with 64 GB RAM, and supported by 2 GeForce RTX 2080 GPUs.

### 4.1. Dataset

The evaluation of our approach is conducted on two public datasets: BSDS500 [8] and NYUD [9]. BSDS500 comprises 200 training images, 100 validation images, and 200 testing images. The PASCAL VOC Context dataset [37] is included in BSDS500 as an additional training set. The ground truth is the averaged annotation labeled manually by multiple human annotators. NYUD consists of 1449 pairs of aligned RGB and depth images split into 381 training, 414 validation, and 654 testing images. To augment the training data size, all the training or validation images in both datasets undergo random flipping, scaling, and rotating operations. Both BSDS500 and NYUD are popular and widely used for edge detection tasks due to their rich annotations, diversity of scenes and objects, and challenging edge cases. Also, standardized evaluation metrics are provided for assessing the performance of edge detection algorithms:

- *Precision*: The fraction of the correctly predicted edges (true positives) among the all-predicted edges.
- *Recall (also known as sensitivity)*: The fraction of the correctly predicted edges (true positives) against the all-ground-truth edges.
- *Average Precision (AP)*: Among the all-predicted edges, AP is the averaged value for all the images.
- *F-measure*: Reflects the relationship between the system’s precision and recall values (Equation (7)).
  (7) $$F_{measure}=2\cdot \frac{Precision\cdot Recall}{Precision+Recall}$$
- *Optimal Dataset Scale (ODS)*: Calculates the averaged precision–recall curves for all the images in the dataset. The ODS score is the best F-measure score using a global threshold. The ODS measures the quality of the edge detection for the entire dataset.
- *Optimal Image Scale (OIS)*: Calculates the best threshold and corresponding F-measure for each image. The OIS score is the averaged F-measure score for all the images. The OIS measures the quality of the edge detection for each individual image.

The backbone network is based on the pre-trained VGG16 model serving as the network initialization. Regarding the loss functions, the hyper-parameter λ is set as 1.1 and 1.2 for BSDS500 and NYUD, respectively. The SGD optimizer is employed to train the network for 40,000 iterations on both datasets, with a batch size of 10 for all experiments. The initial learning rate, momentum, and weight decay are set to $10^{-6}$, 0.9, and $2\times 10^{-4}$, respectively. The learning rate undergoes a 10-fold decrease after every 10,000 iterations.

### 4.2. Ablation Study

We assessed the quality of individual SOs and $MASE_{1}^{k}$ maps through training and testing on BSDS500. Table 1 presents the detection performance of the individual $so_{i}$ without fusion. For example, when i=1, it means that $so_{1}$ is the final edge map. Generally, the qualities of $so_{i}$ from later stages are superior due to the reduction in local details and noise. $so_{4}$ stands out as the most accurate, striking a balance between global contours and fine details. Table 2 provides the comparison among the hierarchical $MASE_{1}^{k}$ maps, where a larger *k* indicates edges endorsed by more global views.

It can be observed from both Table 1 and Table 2 that most MASE maps outperform SOs. This shows the validity of the MASE framework in feature extraction. $MASE_{1}^{5}$ and $MASE_{2}^{5}$ exhibit the best edge quality by encompassing salient edges across almost all the SOs, which shows that edges endorsed by more global view SOs have better performance. The ODS of $MASE_{2}^{5}$ reaches 0.778, only 0.024 less than the human performance. The following experiments show that combining the MASE and SOs can further improve the performance, and it reaches the best when all the MASEs and SOs are fused. We can conclude that the components introduced in our method are valid in boosting edge detection performance.

During the training stage, the loss of the side outputs (SOs) and MASE maps is taken into consideration. To examine the effectiveness of the SOs and MASE maps for providing guidance and deep supervision for network learning, several training variants are built, and their qualitative results on BSDS500 are illustrated in Table 3. Although the ODS score (0.804) for supervising fusion exceeds the human performance (0.803), it ranks lowest among the deep supervision variants. Supervision on fusion, SOs, and MASE maps yields superior results, validating our design approach. Notably, supervision on SO maps and fusion performs closely to the best variant. This result can be attributed to the fact that MASE maps are derived from SO maps, leading to data duplication and limiting the effectiveness of applying loss functions solely on MASEs.

### 4.3. Comparison with State of the Arts

**Figure 5:**
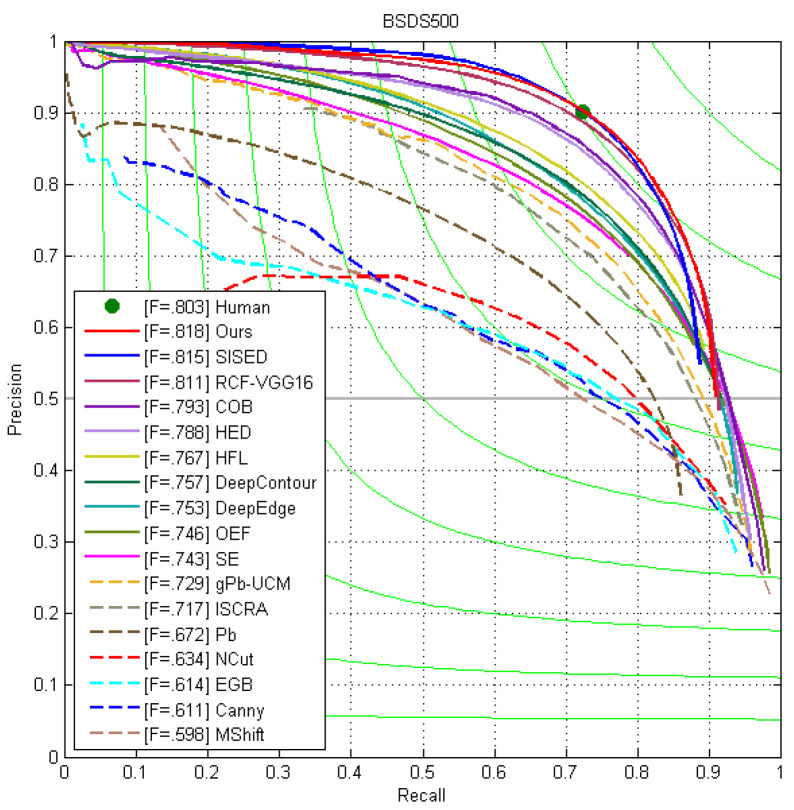
The precision–recall curves of our method and other works on the BSDS500 test set.

We conducted comparative experiments on the BSDS500 dataset first, and the evaluation results are depicted in Figure 5. The performance of the human eye in edge detection is denoted as a 0.803 ODS F-measure. Our method surpassed human performance, and its precision–recall curve surpasses many, including HED and RCF [6,19]. These results underscore the effectiveness and robustness of MASE-based edge features. Table 4 reveals that ours ranks at the top for the ODS, OIS, and AP scores, which are 0.818, 0.837, and 0.848, respectively.

**Figure 6:**
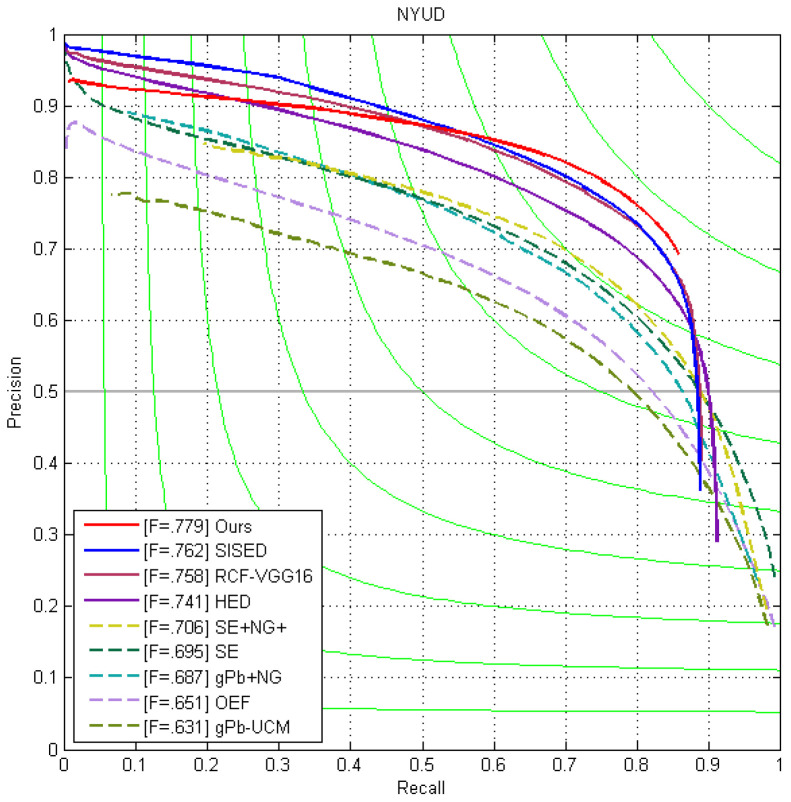
The precision–recall curves of our method and other works on the NYUD test set.

Similarly, we performed comparisons on the NYUD dataset, and Figure 6 illustrates that the precision–recall curve of the MASE outperforms the others. In Table 5, ours consistently ranks higher than all the other methods, with 0.779, 0.792, and 0.775 for the ODS, OIS, and AP, respectively. Compared with the works with the similar network settings, our ODS score is 0.038, 0.022, and 0.017 higher than HED, RCF, and SISED, respectively.

### 4.4. Subjective Evaluations

**Figure 7:**
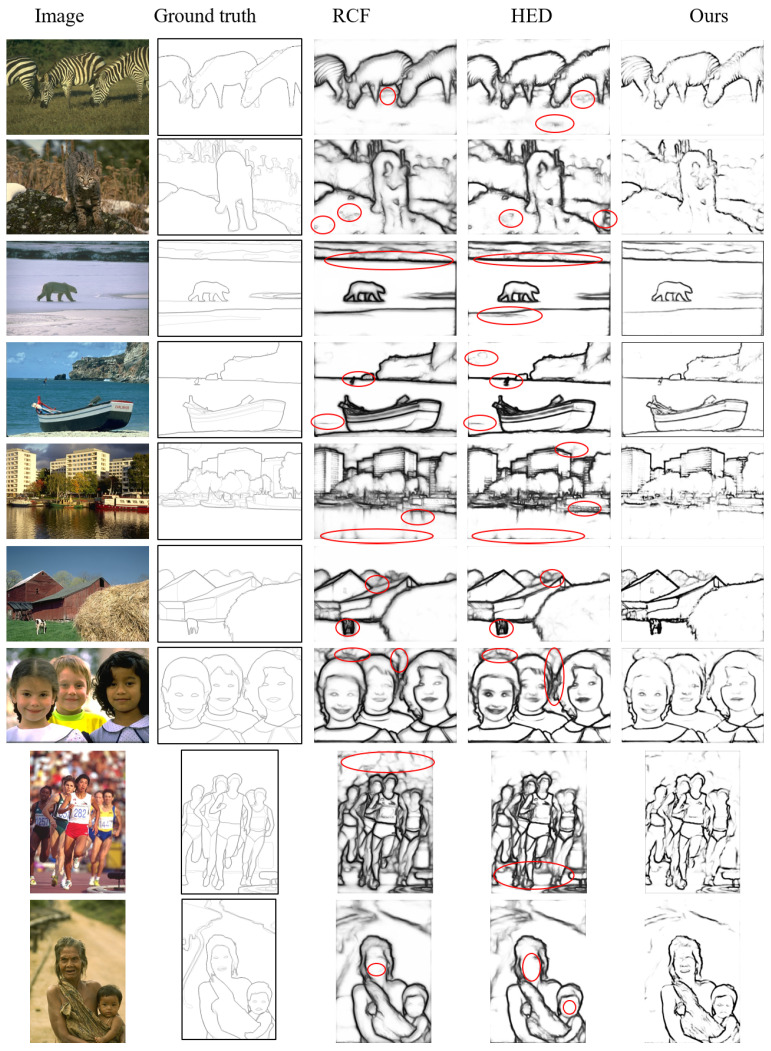
Comparison of some edge detection results on the BSDS500 test set. All the results are raw edge maps computed with a single-scale input before non-maximum suppression. The defects are circled in red.

Figure 7 presents nine example images from the BSDS500 test sets, alongside their corresponding ground-truth images and the final results from various approaches for subjective evaluation. The contents of the image are diverse, including animals in wild scenes, building structures, landscaping, human faces, and sport activities groups. They pose some challenging tasks for edge detection because such a boundary drawing task is even difficult for humans. Even though the other two approaches present reasonable results, our results show much better performance in terms of the noise volume and edge sharpness. The red circles highlight the defects in the results of the RCF and HED methods. Overall, the results from RCF exhibit thicker edge boundaries and more unwanted details compared to the ground truth. This is primarily attributed to the design effort of RCF, which incorporates more fine details into the SOs. For instance, in the fifth row, the cloud and object reflections are retained in the result. In the eighth row, the edges from background people are picked up, which are not part of the ground truths. However, some important details are still missing. For the image in the bottom row, the facial details are not detected. In general, the edges in the RCF results appear blurry and thick with many unwanted local details.

The results from HED contain considerable cluttered noise. For example, in the zebra (the first row) and mountain lion images (the second row), more pixels of grass and clutter on the ground are picked up in the edge results. Similarly, some non-important cloud and water patterns are also treated as object contours. Similar to RCF, for the image in the bottom row, the necessary facial details are missing in the HED methods. In general, the edges from the proposed approach are clearer, thinner, and superior from a human visual perception standpoint.

## 5. Discussion and Future Work

The performance gains observed in the comparative experiments and subjective observation primarily stem from the inclusion of high-quality additional Mutually Agreed Salient Edge (MASE) maps and the improved side outputs (SOs). Ablation studies demonstrate that the MASE generally outperforms original SOs, with $MASE_{2}^{5}$ alone achieving performance levels comparable to human performance. This highlights the effectiveness of our NHP-based operation in edge detection. Moreover, the integration of MASE maps plays a crucial role in guiding the deep supervision during training, resulting in the improved quality of SOs. Even in a variant framework utilizing SOs only, employing the same settings as HED and RCF, our method achieves an F-measure ODS of 0.815, surpassing HED and RCF by 0.027 and 0.004, respectively. This underscores the positive impact of incorporating MASE maps in enhancing the quality of SOs.

The concept of mutual agreements among different views offers valuable insights for achieving ground truth in edge detection. However, according to our observation, instances of true edges may appear in one side output (SO) but are missing in the final results when compared with labeled data. Those missing edges are indeed the disagreed salience. Therefore, the exploration of Disagreed Salient Edges (DSEs) also holds promise for future study. By leveraging MASE maps, we can extract DSEs through subtraction operations, SO−MASE, where each MASE map represents the agreed edges, and the remainder are disagreements. This operation can be implemented within an additional subtraction layer after the NHP operation in the network, with positive weights assigned during fusion if the DSE maps prove beneficial. All weights are learned during training within the end-to-end framework, pointing toward a promising direction for future research.

Our approach is a generic solution, as the process of generating MASE or DSE maps remains independent of the backbone network. By replacing the backbone network, our framework can be applied to different backbone networks, as seen in practices such as RCF [19], where VGG is replaced with a skip connection in ResNet-52. Detaching the current network and plugging in a new one can demonstrate the merits of our framework in future studies. However, integrating advanced backbone networks into our framework poses challenges, especially for transformer-based encoder–decoder networks. As evidenced in the related work, directly integrating U-Net with HED yielded a poorer performance than expected [25]. Thus, the seamless integration of our NHP-based MASE framework with advanced backbones requires substantial efforts in future studies.

## 6. Conclusions

In conclusion, the existing mainstream edge detection methods suffer from the limitation of applying a single weight indiscriminately to each feature side output (SO) during the fusion process, resulting in noisy edges or missing boundaries. To address this challenge, we propose a new normalized Hadamard-product (NHP)-based operation layer within a deep network for edge detection. This innovative approach introduces Mutually Agreed Salient Edge (MASE) maps by multiplying SOs from the backbone network, fostering agreement among features across different scales while suppressing weak signals. The introduction of MASE maps provides a richer hierarchical structure that categorizes edge features into varying levels of importance, effectively discerning between local and global edges. The advantages of this method include the enhanced granularity of edge maps during fusion, enabling the selective inclusion of crucial details from each edge map and thereby improving edge quality and detection accuracy without adding complexity. Ablation studies and comparative experiments further underscore the efficacy of our proposed approach. Our experiments demonstrate that the NHP-based MASE maps enhance performance, with the ODS score reaching 0.818 on the BSDS500 dataset, surpassing human performance (0.803). This achievement underscores the capability of our approach to excel in edge detection tasks and achieve state-of-the-art performance. Furthermore, as evidenced in our work, agreements play crucial roles in promoting salient edges while acknowledging that disagreements may also contribute positively to the detection task. Therefore, investigations into the significance of disagreements is worthy of further study.

## Figures and Tables

**Table 1:** Performance of individual $so_{i}$ on BSDS500, where *so*_4_ achieves highest ODS and OIS scores, and *so*_3_ has the best AP.

${\mathrm{so}}_{i}$	ODS	OIS	AP
$so_{1}$	0.608	0.634	0.617
$so_{2}$	0.713	0.733	0.709
$so_{3}$	0.761	0.781	**0.778**
$so_{4}$	**0.770**	**0.784**	0.763
$so_{5}$	0.747	0.754	0.702

**Table 2:** Performance of individual $MASE_{j}^{k}$ on BSDS500BSDS500, where $MASE_{2}^{5}$ achieves the best ODS and OIS scores.

${\mathrm{MASE}}_{j}^{k}$	ODS	OIS	AP
$MASE_{1}^{2}$	0.692	0.714	0.711
$MASE_{1}^{3}$	0.736	0.757	0.742
$MASE_{1}^{4}$	0.762	0.783	0.752
$MASE_{1}^{5}$	0.775	0.789	0.717
$MASE_{2}^{3}$	0.748	0.769	**0.777**
$MASE_{2}^{4}$	0.768	0.79	0.771
$MASE_{2}^{5}$	**0.778**	**0.793**	0.731
$MASE_{3}^{4}$	0.772	0.79	0.768
$MASE_{3}^{5}$	0.763	0.78	0.723
$MASE_{4}^{5}$	0.741	0.76	0.701

**Table 3:** Effectiveness of deep supervision on BSDS500, where the deep supervision on fusion, where SO and MASE maps performs the best.

Overall Loss Function (*L*) for Training Variants	ODS	OIS	AP
supervision on fusion only $L_{fuse}$	0.804	0.821	0.819
supervision on fusion and SO maps $\sum L_{s}+L_{fuse}$	0.815	0.832	0.820
supervision on fusion and MASE maps $\sum L_{m}+L_{fuse}$	0.807	0.812	0.837
supervision on fusion, SO maps, and MASE maps $\sum L_{s}+\sum L_{m}+L_{fuse}$	**0.818**	**0.837**	**0.848**

**Table 4:** The comparison with some methods on BSDS500.

Method	ODS	OIS	AP
Canny [10]	0.611	0.676	0.520
EGB [38]	0.614	0.658	0.564
MShift [39]	0.598	0.645	0.497
OEF [40]	0.746	0.770	0.815
HFL [41]	0.767	0.788	0.795
$N^{4}$-Fields [3]	0.753	0.769	—
DeepContour [5]	0.757	0.776	0.790
DeepEdge [2]	0.753	0.772	0.807
RDS [42]	0.792	0.810	—
CEDN [43]	0.788	0.804	—
HED [6]	0.788	0.808	0.840
RCF [19]	0.811	0.830	0.846
SISED [44]	0.815	0.835	0.839
**Ours**	**0.818**	**0.837**	**0.848**

**Table 5:** The comparison with some methods on NYUD.

Method	ODS	OIS	AP
gPb-UCM [8]	0.631	0.661	0.562
OEF [40]	0.651	0.667	0.653
gPb-NG [45]	0.687	0.716	0.629
SE [18]	0.695	0.708	0.719
SE + NG [46]	0.706	0.734	0.549
HED [6]	0.741	0.757	0.749
RCF [19]	0.757	0.771	0.749
SISED [44]	0.762	0.786	0.752
**Ours**	**0.779**	**0.792**	**0.755**

## Data Availability

The raw data supporting the conclusions of this article will be made available by the authors on request.
